# Treatment of Tourette Syndrome With Attention Training Technique—A Case Series

**DOI:** 10.3389/fpsyt.2020.519931

**Published:** 2020-09-29

**Authors:** Anja Schaich, Valerie Brandt, Alena Senft, Christian Schiemenz, Jan-Philipp Klein, Eva Faßbinder, Alexander Münchau, Daniel Alvarez-Fischer

**Affiliations:** ^1^ Department of Psychiatry and Psychotherapy, University of Lübeck, Lübeck, Germany; ^2^ Department of Psychology, Centre for Innovation in Mental Health, University of Southampton, Southampton, United Kingdom; ^3^ Institute of Systems Motor Science, University of Lübeck, Lübeck, Germany

**Keywords:** Gilles de la Tourette syndrome, tic, attention training technique, metacognitive therapy, psychotherapy

## Abstract

The existing therapeutic strategies of Tourette syndrome (TS) do not lead to sufficient improvement in a significant number of patients. Recently published studies show that paying attention to tics increases whereas directing attention away decreases tic frequency. The aim of the present case series in three patients with TS was to investigate the effect of attention training technique (ATT) on TS symptoms. ATT is a technique derived from metacognitive therapy that aims on training patients to consciously (re-)focus their attention away from themselves. Friedman’s chi-square test indicated a trend regarding the reduction of tic frequency and tic severity and a significant reduction of positive metacognitions from pre-baseline to follow-up. Reliable Change Indices (RCIs) are given for each measure and patient. Given the small number of patients, further studies including randomized controlled trials appear warranted.

## Introduction

Tourette syndrome (TS) is a common neuro-psychiatric disorder with a prevalence of 0.3 to 0.7% (1) and potentially detrimental impact on patients’ quality of life (2). Transient tics occur during childhood with a higher prevalence of up to 15–20%. Factors predicting whether tics persist or disappear in adulthood have not been identified to date. Yet, it has clearly been demonstrated that paying attention to tics can transiently increase tic frequency, whereas directing attention away can transiently reduce both tic frequency and severity (3–5).

Behavioral therapy is currently recommended as first-line treatment for tics according to the European and American clinical guidelines for TS (6, 7). The gold standard clinical intervention is comprehensive behavioral intervention for tics (CBIT) including habit reversal therapy (HRT) with the core components awareness training and the acquisition of a competing response that is incompatible with the tic. CBIT appears to be as effective as antipsychotics but is helpful only in about 40% of adults and 53% of children (8) with TS. Thus, a significant number of patients do not respond. Alternative treatment options are therefore needed. Also, recent publications show that paying attention to tics increases tic severity when tics are not actively suppressed, while paying attention to a task decreases tics (3–5, 9). Brandt *et al*. demonstrated that tic frequency was significantly higher when patients watched themselves in a mirror compared to baseline (alone in a room without a mirror) (5). Interestingly, when patients were examined watching a video showing themselves not ticcing, tic frequency was reduced compared to baseline (5). These finding have been corroborated in a follow-up study, where additional conditions were tested including watching own tics in a live video-feedback, in a previously recorded video, thinking about situations that can trigger tics and thinking about specific, non-tic related stimuli (distraction condition) during both free ticcing and tic suppression states (3, 4). The results of this study showed that paying attention to own tics was particularly problematic when tics were not suppressed. On the other hand, paying attention to other stimuli was not helpful when tics had to be suppressed, as is the case during HRT (3, 4). In another study, patients performed rhythmic finger movements triggering a unique visual color stimulus. Patients were asked to monitor and remember their finger actions, the external colors caused by their actions, or their tics. During a “free ticcing” condition patients had significantly fewer tics when attending to finger movements, or to the ensuing colors, compared to the condition where they attended to their tics (4). These data imply that shifting attention away from tics significantly reduces tic frequency (4). Another recent study supports this finding indicating that thinking about tics triggers tics in TS and chronic tic disorder (9).

There is evidence that the functional connectivity between the right caudate nucleus, as well as the right thalamus and left putamen with the precuneus, i.e., connectivity within the default mode network involved in processes related to self-awareness including self- reference (10) is increased in patients with TS (11). This may represent a neural correlate of increased self-referential and self-centered thinking related to tics implying that patients have difficulties to shift attention particularly with respect to tic related processes.

Measures allowing to facilitate shifting attention away from these tic related processes may therefore be very helpful in reducing tic frequency. First pilot reports using mindfulness-based therapy to reduce tics support this hypothesis (12). An approach based on shifting attention away from tics and urges could therefore be an effective treatment for patients with TS.

Attention training technique (ATT) developed by Wells (13) is an approach that is based on training patients to consciously (re-)focus their attention away from themselves. During ATT patients are instructed to regularly practice an external auditory attention exercise including selective attention, attention switching and divided attention to auditory stimuli. This exercise aims at diminishing the focus on internal stimuli and events including tics and increasing the meta-cognitive control of attention. ATT was developed as part of metacognitive therapy and been shown to reduce symptoms in depression, panic disorder, and social anxiety as a stand-alone intervention (14).

Based on previous evidence (3–5, 9) that tics can be modulated by attention, the aim of the present clinical case series was to explore whether ATT can reduce tics and enhance quality of life in patients with TS.

## Methods

### Study Design

**Figure 1:**
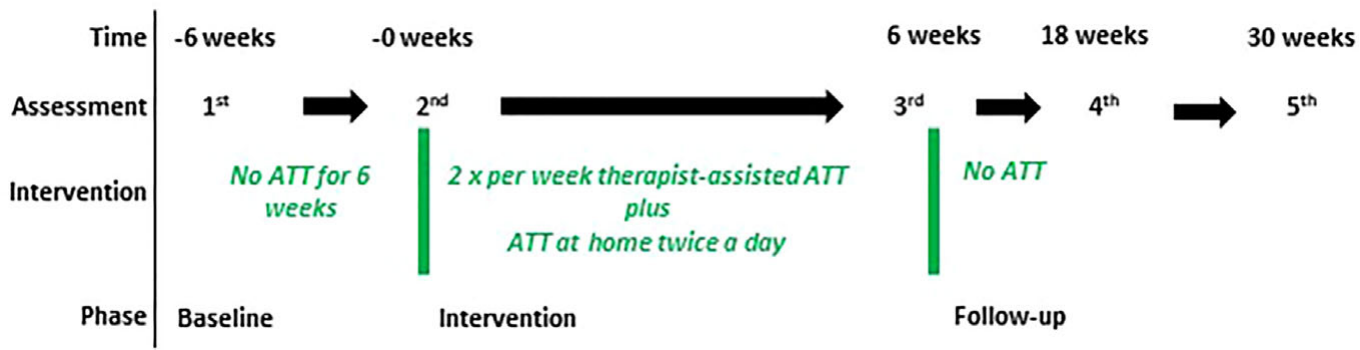
Study design. The figure shows the design of the case series. Patients (n=3) were assessed using the Yale Global Tic Severity Scale (YGTSS), Gilles de la Tourette syndrome-quality of life scale (GTS-QoL), Rush Modified Video-Based Rating Scale, Premonitory Urge for Tics Scale (PUTS), Metacognitions Questionnaire (MCQ30), and Thinking about Tic (THAT) at each assessment. During the study medication was unchanged. ATT started after baseline (0 weeks). After the intervention (6 weeks), patients discontinued ATT and were again assessed 18 and 30 weeks after the start of the intervention as follow-up.

Three patients with TS were recruited within the outpatient center of the Department of Psychiatry and Psychotherapy of the Lübeck University Clinic in Germany. Participants were considered eligible if they were aged 18 or above and had 1) a main diagnosis of TS [according to Diagnostic and Statistical Manual of Mental Disorders, 5th Edition (DSM-5) criteria] (15), 2) had read and signed an informed consent form, 3) were able and willing to participate reliably in therapy and assessment procedures. Exclusion criteria were 1) lifetime diagnosis of a psychotic or bipolar disorder, 2) intellectual deficits (IQ < 85), 3) acute suicidality, 4) a main diagnosis other than TS that requires prioritized treatment, and 5) acute substance dependency (according to DSM-5) that requires detoxification treatment. The willingness to engage in a new type of behavioral therapy was spontaneously the highest in the addressed patients, which might explain a selection bias. All participants were diagnosed by AM and DAF using the Structural Clinical Interview for DSM-IV (SCID I and II) (16) and received a 6-week-ATT program (10 sessions plus homework twice a day) after a 6-weeks no-treatment baseline. Subsequently, patients stopped performing ATT at home and attended no further therapy sessions. Assessments took place before and after baseline, after the intervention phase, and at 18 weeks and 30 weeks after the start of treatment as follow-up (**Figure 1**). Medication was unchanged during the whole period and is given in the supplements. All individuals gave written informed consent and the local ethics committee approved the study (AZ 16-097).

### Patient 1

Patient 1 (red curve) was a 35-year old woman with a 24-year history of TS. Current tics included winking, nasal flare, and raising the corners of her mouth. She has had vocal tics in the past. She suffered from comorbid generalized anxiety disorder, attention deficit hyperactivity disorder (ADHD) and described obsessive compulsive symptoms but did not meet the criteria for obsessive compulsive disorder (OCD).

### Patient 2

Patient 2 (blue curve) was a 25-year old woman with a 20-year history of TS. Current tics included sudden head movements sideways or backwards, stretching her leg and vocal tics (shouting words). She suffered from comorbid ADHD and OCD and had been successfully treated for drug addiction and post-traumatic stress disorder.

### Patient 3

Patient 3 (green curve) was a 43-year old woman with a history of TS since early childhood. Current tics included eye blinking, moving the corner of her mouth, and raising one shoulder. As a child, she underwent psychotherapy for her tic disorder. She suffered from comorbid major depressive disorder.

### Attention Training Technique

ATT, one of the key strategies of the metacognitive therapy developed by Wells (13), consists of three phases (selective attention, rapid attention switching, and divided attention) and lasts approximately 12 min. During ATT, patients are instructed to focus their attention on at least five competing auditory stimuli. In the first phase, patients are instructed to pay attention to each stimulus in turn and to bring their attention back to that stimulus should they be distracted. During the second phase, they are asked to rapidly switch their attention from one stimulus to another and in the third phase they are instructed to expand their attention and to perceive all stimuli simultaneously. A more detailed description of the ATT rationale and introduction (13, 17) is given in the supplements. The ATT rationale was introduced to the patients, followed by a therapist-guided practice of the technique. ATT was conducted as previously described (14). ATT was implemented following the baseline period. The first ATT-session was scheduled with a duration of 45 min and consisted of presenting the ATT rationale followed by therapist-guided practice of the technique. During the following 6 weeks, patients kept practicing ATT twice a day with a provided audio file (see **Supplemental Material**) and received two therapist-guided ATT-sessions per week. At the beginning of each in-session ATT-training, the therapist checked the homework assignment (using a diary kept by the participant) and ensured that the patients practiced ATT correctly and regularly. All participants completed all therapy sessions and practiced ATT at least once each day.

### Clinical Measures

All outcomes were assessed by the use of widely accepted and validated scales (9, 16–20). All assessments were conducted at the Centre for Brain, Behavior, and Metabolism (CBBM) at the University Clinic Lübeck, by a trained psychologist (VB) who was independent of the team delivering the therapy sessions. Assessments took place 6 weeks before the first session, immediately before the first session and within a week after the last session, as well as 18 weeks and 30 weeks after the start of treatment as follow-up.

*Tic frequency and severity* was assessed using the Yale Global Tic Severity Scale (YGTSS50) (18) and the Modified Rush Videotape-Rating-Scale (19). The YGTSS50 is a clinician-rated measure of tic severity. The motor and phonic tics are rated separately on a 0–5 scale across five dimensions (number, frequency, intensity, complexity, and interference). A Total Tic score (range 0–50) can be calculated and was used as outcome in this study. Using the Modified Rush-Videotape-Rating-Scale two independent raters (C.S., K.H.) blinded to the study and study design scored the number of phonic and motor tics the patients displayed in a room with no examiner present (5 min.). Inter-rater reliability for the video rating was high (*R* = 0.96, *p* < 0.001). Pre- and post-treatment video order was randomized and blinded to the raters.

*The urge to tic* was measured using the Premonitory Urge for Tics Scale (PUTS) (20). The PUTS consists of ten items rated on a four-point scale (1= “not at all true”, 4 = “very much true”; range 10–40) measuring sensory and mental phenomena associated with premonitory urges. Six items of the PUTS address itchiness, energy, pressure, tense feeling, incomplete, or a “just not right” feeling before performing a tic. Four additional items assess how often these phenomena are experienced before a tic, if they are reduced after the performance of a tic and if subjects are able to stop the tics (e.g., “I am able to stop my tics, even if only for a short period of time”).

*Quality of life* was assessed using the Gilles de la Tourette syndrome-quality of life scale (GTS-QoL) (21). The GTS-QOL consists of 27-item scale assessing four subscales (psychological, physical, obsessive-compulsive, and cognitive). Scores for the four subscales are generated by summing items and normalizing total scores to a 0–100 range with higher scores indicating worse health-related quality of life.

*Positive and negative metacognitions* (thoughts about thinking) were assessed using the Metacognitions Questionnaire (MCQ-30) (22). The MCQ-30 is a 30-item questionnaire that assesses five factors of metacognition: positive beliefs (e.g., “worrying helps me to avoid problems in the future”), negative beliefs (e.g., “my worrying is dangerous for me”), cognitive confidence, cognitive self-confidence, and need to control thoughts. Items are rated on a four-point scale (do not agree, agree slightly, agree moderately, agree very much) and responses are summed up to produce total scores for each area.

*Thinking about tics* was assessed using the Thinking-about-Tics-Inventory (THAT) (9). The THAT consists of 22 items rated on a 3-point scale (1= always, 2 = sometimes, 3 = never). Patients are instructed to indicate whether specific thoughts have triggered tics. Thoughts that are assessed are related to interference (e.g., “wondering if your tics will interfere with your activities”), anticipation (e.g., “anticipating that you might tic”), and permission (“knowing that you have permission to tic”).

### Statistical Analysis

All data are given as absolute values. Data analyses were conducted using IBM SPSS Statistics 25 (SPSS Inc., USA). With the non-parametric Friedman-Test for repeated measurements, we analyzed differences between pre- and post-treatment. Significance level was set at 0.05. We used the Reliable Change Indices (RCIs) to indicate change within the individual participants. Values equal/higher than 1.96 or equal/smaller than 1.96 are representative of a reliable change at a 95% confidence level (*p*-value = 0.05) (23). Spearman correlations were used to assess the relationship between tic frequency and negative metacognitions. As this study was conducted with an exploratory purpose for hypothesis generation we refrained from correcting for multiple comparisons in order to prevent accumulation of type II error.

## Results

### Feasibility

All patients completed the study.

### Effectiveness in Tic Reduction

**Figure 2:**
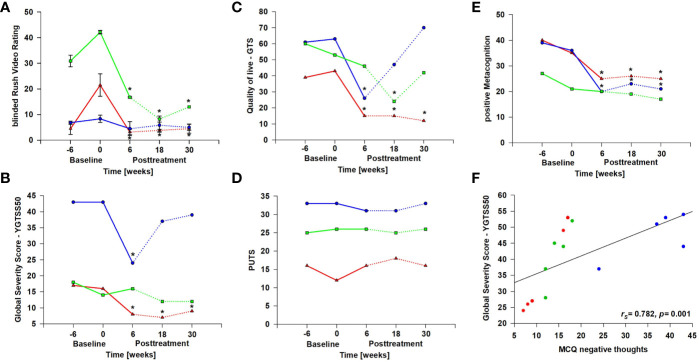
Study results. Absolute values are given for each patient (patient 1 = red, patient 2 = blue, patient 3 = green). Dashed lines indicate post intervention time points. * Reliable Change Indices (RCIs) indicating that individual change scores are statistically significantly greater than expected by chance. **(A)** Rush Video-Based Rating Scale: in the Rush Video scale the subjective improvement was verified by two blinded raters to whom videos were presented in a random manner. Inter-rater correlation was r_S_=0.96 (**Figure S1**). **(B)** YGTSS50: Yale Global Tic Severity Scale. **(C)** GTS-QoL: Gilles de la Tourette syndrome-quality of life scale. (**D**) PUTS: Premonitory Urge for Tics Scale. **(E)** MCQ30 positive metacognitions: Metacognitive Questionnaire, subscale “positive beliefs about worry” (such as “worrying helps me to avoid problems in the future”). **(F)** shows the correlation between Tic severity as measured with the YGTSS50 and MCQ30 negative metacognitions: Metacognitive Questionnaire, subscale “negative beliefs about worry” (such as “worrying is dangerous for me”).

There was a trend regarding tic reduction from pre-baseline to follow-up both measured by the YGTSS [YGTSS50: Friedman *χ^2^*(4) = 8.69, *p* = .069] and the Rush Video-based Rating scale [Friedman *χ^2^*(4) = 9.42, *p* = .051] (**Figures 2A, B**). Reliable change indices (RCIs) of the Rush Video-Based Rating scale indicate that a clinically significant reduction in tic frequency was achieved in patient 1 and 2 at post-treatment compared to pre-treatment and remained stable during the follow-up period (**Table 1**). When individual values of Rush Video-Rating at baseline were set as 100%, changes after intervention are significant for all post-treatment measurements (one-way ANOVA repeated measurements with *post-hoc* Holm-Sidak test, see **Supplemental Material**). Reliable change indices (RCIs) of the YGTSS50 indicated a meaningful reduction in patient 1 and patient 2 at post-treatment compared to pre-treatment, but was only maintained during follow-up by patient 1 (**Table 1**).

**Table 1:** Reliable Change Indices (RCIs) of the measurements.

	Score		RCI	Reliable change (RCI > 1.96)
**Rush Video-Based Rating Scale**				
Patient 1				
Pre-treatment	22			
Post-treatment	3		5.43	Yes
3-Month follow-up	4		5.14	Yes
6-Month follow-up	5		4.86	Yes
Patient 2				
Pre-treatment	8			
Post-treatment	5		0.86	No
3-Month follow-up	6		0.57	No
6-Month follow-up	5		0.86	No
Patient 3				
Pre-treatment	42			
Post-treatment	17		7.14	Yes
3-Month follow-up	8		9.71	Yes
6-Month follow-up	13		8.29	Yes
**Yale Global Tic Severity Scale (YGTSS50)**				
Patient 1				
Pre-treatment	16			
Post-treatment	8		2.45	Yes
3-Month follow-up	7		2.76	Yes
6-Month follow-up	9		2.15	Yes
Patient 2				
Pre-treatment	43			
Post-treatment	24		5.83	Yes
3-Month follow-up	37		1.84	No
6-Month follow-up	39		1.23	No
Patient 3				
Pre-treatment	14			
Post-treatment	16		−0.61	No
3-Month follow-up	12		0.61	No
6-Month follow-up	12		0.61	No
**GTS-Quality of Life Questionnaire (GTS-QOL)**				
Patient 1				
Pre-treatment	43			
Post-treatment	15		1.97	Yes
3-Month follow-up	15		1.97	Yes
6-Month follow-up	12		2.17	Yes
Patient 2				
Pre-baseline*	61			
Post-treatment	26		2.45	Yes
3-Month follow-up	47		0.98	No
6-Month follow-up	70		−0.63	No
Patient 3				
Pre-treatment	53			
Post-treatment	46		0.49	No
3-Month follow-up	24		2.03	Yes
6-Month follow-up	42		0.77	No
**Metacognitive Questionnaire (MCQ 30): subscale positive metacognitons**				
Patient 1				
Pre-treatment		35		
Post-treatment		25	2.16	Yes
3-Month follow-up		26	1.95	Yes
6-Month follow-up		25	2.16	Yes
Patient 2				
Pre-treatment		36		
Post-treatment		20	3.46	Yes
3-Month follow-up		23	2.81	Yes
6-Month follow-up		21	3.25	Yes
Patient 3				
Pre-treatment		21		
Post-treatment		20	0.22	No
3-Month follow-up		19	0.43	No
6-Month follow-up		17	0.87	No

*As patient 2 did not complete the GTS-QOL at baseline, pre-baseline data was used to calculate the RCI in this patient

### Effectiveness in Self-Assessment-Based Questionnaires

In the GTS-QoL (**Figure 2C**), all patients reported meaningful improvement of their quality of life at post treatment and/or at 18-week follow-up compared to pre-treatment as indicated by RCIs (**Table 1**). However, the improvement in quality of life was stable during the follow-up period only in patient 1. Friedman *χ^2^*-tests were not significant. Interestingly, neither the urge to tic as determined with the PUTS (**Figure 2D**) nor thoughts triggering tics (THAT, see **Supplemental Material**) changed over the course of the study.

### Thoughts About Thinking

The results of the MCQ indicated a reduction of positive metacognitions (such as “worrying helps me to avoid problems in the future”) [Friedman *χ^2^*(4) = 10.64, *p* = .03] (**Figure 2E**). There was a significant reduction from pre-treatment to post-treatment (Dunn-Bonferroni: *z* = 3.17, *p* = .014) as well as from pre-treatment to 30-week follow-up (Dunn-Bonferroni: *z* = 3.50, *p* = .007). As indicated by the RCIs patient 1 and patient 2 experienced a reduction in metacognitions at post treatment and during the follow-up-period (**Table 1**). Negative metacognitions correlated significantly with severity of tics measured by the YGTSS50 (*r*_s_ = .782, *p* = .001, **Figure 2F**).

## Discussion

In the present case series, ATT was not only feasible and well accepted by the patients but seems to have the potential to reduce tic frequency and severity as well as to improve quality of life as indicated by RCIs. Interestingly, the improvement of the patient with the highest TS severity (patient 2) did not persist during the follow-up phase when measured by the YGTSS50. Similar to CBIT (24) it appears that for ATT in patients with TS booster sessions after 3-months might be necessary. According to the present data it is not possible to identify patterns that might predict response to the technique due to small sample size. With the reported series we first aimed to show the proof-of-principle and the feasibility of ATT in TS. 

ATT is a therapeutic intervention targeting meta-cognitive processes. It is currently unclear how meta-cognition may be related to tics. Our results indicate a correlation between negative metacognitions and tic frequency. It is tempting to speculate that regarding own thoughts as uncontrollable and dangerous might contribute to tics. Further studies will elucidate whether this is a relevant underlying mechanism.

To our knowledge, this is the first behavioral intervention that is not related to awareness toward the urge to tic and the generation of alternative coping strategies in respond to urges. In the participants of this study, the urge to tic remained unaltered. This is in line with results from previous studies in TS showing that behavior therapy fails to change premonitory urge severity (25) independent of the treatment outcome (25). These results support doubts that habituation is the therapeutic process underlying the effectiveness of behavior therapy for TS (25). However, it is also possible that changes in urges over time are not picked up by the PUTS.

Given the small number of participants and the one-armed study design, results must be interpreted with caution. The study design with two baseline assessments takes natural tic fluctuation into account but does not control for it. Tic fluctuation during baseline interval however was small and does not seem high after intervention either. TS is often accompanied by other mental disorders and the patients included in our study also suffered from comorbid disorders such as anxiety disorders and depression. ATT has proven effective for treating anxiety and depression (13, 14, 26) and it is not possible to disentangle the degree, to which the changes observed in this study are related to the effect of ATT on the comorbid disorders. All participants in our study were female despite a gender distribution in favor for males (m/f 2:1 in adults) (1). Although sex did not emerge as a moderator of response for behavioral treatment in TS in previous studies [at least for the CBIT (27)], females are a special subgroup of patients with TS and reproducibility in general and especially in males has to be shown. Further studies including randomized controlled trials appear warranted.

## Data Availability Statement

The datasets generated for this study are available on request to the corresponding author.

## Ethics Statement

The studies involving human participants were reviewed and approved by Ethics Committee of the Lübeck University (reference number: AZ 16-097). The patients/participants provided their written informed consent to participate in this study. Written informed consent was obtained from the individual(s) for the publication of any potentially identifiable images or data included in this article.

## Author Contributions

Conception and design of the study: ASc, VB, AM, DA-F. Acquisition and Analysis of data: ASc, ASe, CS, VB, DA-F. Drafting of the Manuscript: ASc, VB, AM, J-PK, EF, DA-F.

## Funding 

DA-F was supported by a grant from the Fritz-Thyssen-Stiftung (10.16.1.019MN). AM is supported by the Deutsche Forschungsgemeinschaft (FOR 2698). ASc received financial support by Land Schleswig-Holstein within the funding programme Open Access Publikationsfonds. Funding bodies played no role in the design of the study, in the collection, analysis and interpretation of data, in the writing of the manuscript and in the decision to submit the manuscript for publication.

## Conflict of Interest

The authors declare that the research was conducted in the absence of any commercial or financial relationships that could be construed as a potential conflict of interest.

## References

[1] Yang J Hirsch L Martino D Jette N Roberts J Pringsheim T The prevalence of diagnosed tourette syndrome in Canada: A national population-based study. Mov Disord (2016) 31(11):1658–63. 10.1002/mds.26766 27548401

[2] Bernard BA Stebbins GT Siegel S Schultz TM Hays C Morrissey MJ Determinants of quality of life in children with Gilles de la Tourette syndrome. Mov Disord (2009) 24(7):1070–3. 10.1002/mds.22487 19306279

[3] Herrmann K Sprenger A Baumung L Alvarez-fischer D Mu A Brandt V Help or hurt ? How attention modulates tics under different conditions. Cortex (2019) 120:471–82. 10.1016/j.cortex.2019.06.016 31491584

[4] Misirlisoy E Brandt V Ganos C Tübing J Münchau A Haggard P The Relation Between Attention and Tic Generation in Tourette Syndrome. Neuropsychology (2015) 29(4):658–65. 10.1037/neu0000161 PMC4484548 25486384

[5] Brandt VC Lynn MT Obst M Brass M Münchau A Visual feedback of own tics increases tic frequency in patients with Tourette’s syndrome. Cognit Neurosci Internet (2015) 6(1):1–7. 10.1080/17588928.2014.954990 25185800

[6] Roessner V Plessen KJ Rothenberger A Ludolph AG Rizzo R Skov L European clinical guidelines for Tourette syndrome and other tic disorders. Part II: Pharmacological treatment. Eur Child Adolesc Psychiatry (2011) 20(4):173–96. 10.1007/s00787-011-0163-7 PMC3065650 21445724

[7] Pringsheim T Okun MS Muller-Vahl K Martino D Jankovic J Cavanna AE Practice guideline recommendations summary: Treatment of tics in people with Tourette syndrome and chronic tic disorders. Neurology (2019) 92(19):896–906. 10.1212/WNL.0000000000007466 31061208 PMC6537133

[8] Capriotti MR Himle MB Woods DW Behavioral Treatments for Tourette Syndrome. J Obs Compuls Relat Disord (2014) 3(4):415–20. 10.1016/j.jocrd.2014.03.007 PMC6150491 30245958

[9] O’Connor K St-Pierre-Delorme MÈ Leclerc J Lavoie M Blais MT Meta-cognitions in Tourette syndrome, tic disorders, and body-focused repetitive disorder. Can J Psychiatry (2014) 59(8):417–25. 10.1177/070674371405900804 PMC4143298 25161066

[10] Cavanna AE Trimble MR The precuneus: a review of its functional anatomy and behavioural correlates. Brain (2006) 129(Pt 3):564–83. 10.1093/brain/awl004 16399806

[11] Ramkiran S Heidemeyer L Gaebler A Shah NJ Neuner I Alterations in basal ganglia-cerebello-thalamo-cortical connectivity and whole brain functional network topology in Tourette’s syndrome. NeuroImage Clin (2019) 24(January). 10.1016/j.nicl.2019.101998 PMC6742843 31518769

[12] Reese H Vallejo Z Rasmussen J Crowe K Rosenfield E Wilhelm S Mindfulness-based stress reduction for Tourette Syndrome and Chronic Tic Disorder: a pilot study. J Psychosom Res (2015) 78(3):293–8. 10.1016/j.jpsychores.2014.08.001 25149879

[13] Wells A Panic disorder in association with relaxation induced anxiety: An attentional training approach to treatment. Behav Ther (1990) 21:273–80. 10.1016/S0005-7894(05)80330-2

[14] Knowles MM Foden P El-Deredy W Wells A A Systematic Review of Efficacy of the Attention Training Technique in Clinical and Nonclinical Samples. J Clin Psychol (2016) 72(10):999–1025. 10.1002/jclp.22312 27129094

[15] American Psychiatric Association Diagnostic and statistical manual of mental disorders : DSM-5 [Internet]. Fifth edition American Psychiatric Publishing: Arlington, VA (2013).

[16] Wittchen HU Fydrich T Wittchen HU Zaudig M SKID: Strukturiertes Klinisches Interview für DSM-IV; Achse I und II. Hogrefe (1997).

[17] Wells A Metacognitive Therapy for Anxiety and Depression. Guilford Press: New York, NY, US (2009).

[18] Storch EA Murphy TK Geffken GR Sajid M Allen P Goodman WK Reliability and validity of the Yale Global Tic Severity Scale. Psychol Assess (2005) 17(4):486–91. 10.1037/1040-3590.17.4.486 16393016

[19] Goetz CG Pappert EJ Louis ED Raman R Leurgans S Advantages of a modified scoring method for the Rush Video-Based Tic Rating Scale. Mov Disord (1999) 14(3):502–6. 10.1002/1531-8257(199905)14:3<502::AID-MDS1020>3.0.CO;2-G 10348478

[20] Woods DW Piacentini J Himle MB Chang S Premonitory Urge for Tics Scale (PUTS): initial psychometric results and examination of the premonitory urge phenomenon in youths with Tic disorders. J Dev Behav Pediatr (2005) 26(6):397–403. 10.1097/00004703-200512000-00001 16344654

[21] Cavanna AE Schrag A Morley D Orth M Robertson MM Joyce E Gilles de la Tourette syndrome-quality of life scale (GTS-QOL): development and validation. Neurology (2008) 71(18):1410–6. 10.1212/01.wnl.0000327890.02893.61 18955683

[22] Cartwright-Hatton S Wells A Beliefs about worry and intrusions: the Meta-Cognitions Questionnaire and its correlates. J Anxiety Disord (1997) 11(3):279–96. 10.1016/S0887-6185(97)00011-X 9220301

[23] Jacobson NS Truax P Clinical significance: A statistical approach to defining meaningful change in psychotherapy research. J Consult Clin Psychol (1991) 59(1):12–9. 10.1037/0022-006X.59.1.12 2002127

[24] Wilhelm S Peterson AL Piacentini J Woods DW Deckersbach T Sukhodolsky DG Randomized trial of behavior therapy for adults with Tourette syndrome. Arch Gen Psychiatry (2012) 69(8):795–803. 10.1001/archgenpsychiatry.2011.1528 22868933 PMC3772729

[25] Houghton DC Capriotti MR Scahill LD Wilhelm S Peterson AL Walkup JT Investigating Habituation to Premonitory Urges in Behavior Therapy for Tic Disorders. Behav Ther (2017) 48(6):834–46. 10.1016/j.beth.2017.08.004 PMC5679290 29029679

[26] Papageorgiou C Wells A Treatment of recurrent major depression with Attention Training. Cognit Behav Pract (2000) 7(4):407–13. 10.1016/S1077-7229(00)80051-6

[27] Sukhodolsky DG Woods DW Piacentini J Wilhelm S Peterson AL Katsovich L Moderators and predictors of response to behavior therapy for tics in Tourette syndrome. Neurology (2017) 88(11):1029–36. 10.1212/WNL.0000000000003710 PMC5384839 28202705

