# Supplementary material for: Treatment of Tourette Syndrome With Attention Training Technique—A Case Series
Source: Front Psychiatry. 2020 Sep 29;11:519931. doi: 10.3389/fpsyt.2020.519931 (PMC7550728; doi:10.3389/fpsyt.2020.519931)
Supplement: Supplementary file 1 [file DataSheet_1.pdf]

Supplementary Material

Supp. 1: Description of the intervention:

ATT was implemented following the baseline period. The first ATT-session was scheduled for a duration of 45 minutes and consisted of presenting the ATT rationale followed by therapist-guided practice of the technique. The rationale explains the role of increased self-focused attention in the maintenance of the tic-disorder.

"*When people have tics, they tend to become highly self-conscious about their urges to perform the tic-behavior. This form of self-preoccupation is problematic because it increases the inner tension and the urge to act out the tic-behavior. You can see this effect in action if you think about your own experiences of the tic-disorder. When you keep focused on the urge to act out the tic and the consequences of acting out the tic, e.g. "what will the others think; ticcing in this situation is highly inappropriate" you can feel your tension rising and you will probably feel how the urge to act out the tic will become more intense and will make you perform the tic more often. However, in situations, in which you are less focused on your urge to tic but more focused on other things, e.g. on vacation, you will feel less urge to tic and perform tics less often. You will learn a technique called Attention Training, which will allow you to prevent or interrupt self-focused attention and reduce your overall level of preoccupation with your tics.*"

Following this rationale, patients were instructed in ATT-practice following the instructions as laid out by Wells (11;12). At least five competing sounds were introduced both inside and outside the therapist room. Patients were to practice the training with their eyes open and focused on a visual fixation point. The ATT practice lasted for approximately 15 minutes. Following the first ATT-session, patients received an audio tape of the ATT training and were asked to practice ATT twice a day.

During the following 6 weeks, patients kept practice ATT twice a day and received a therapist-guided ATT-session once a week. At the beginning of the in-session ATT-training, the therapist checked the

homework assignment and ensured that the patients practiced ATT correctly and regularly. For homework, patients received an audio file containing the exercise.

Medication of patients

Patient 1

Medication was unchanged and consisted of a prescription-free herbal preparation of valeric-acid-zinc-salt, coffee seeds, oats, passion flower.

Patient 2

Medication was unchanged and consisted of methylphenidate, aripiprazole and amitriptyline.

Patient 3

As an adult, she was medicated with clonidine for some time without significant effect. Actual medication remained unchanged and consisted of citalopram and ramipril.

Audio file:

The German version of the ATT audio file is freely accessible following https://www.metakognitivetherapie.de/aufmerksamkeitstraining-att

Figure legend:

Figure S1: Rush Video-based blinded rating. (A) When individual data are set as 100% at baseline, improvement remained significant over the follo-up period of 6 months despite subjective feeling of declining. (B) Interrater reliability of video-based rating is shown by high correlation between independent ratings. Data are given as mean ± S.E.M., one-way ANOVA with repeated measurements and post-hoc Holm-Sidak test.

Figure S2: Thinking about Tics. The graph shows the results of the thinking about Tics inventory (THAT)(18). The results of the item-set about the perceived role of thoughts about tics in eliciting tics remained unaltered by the ATT treatment at any time point.
